# Stress as a Possible Mechanism in Melanoma Progression

**DOI:** 10.1155/2010/483493

**Published:** 2010-05-27

**Authors:** M. Sanzo, R. Colucci, M. Arunachalam, S. Berti, S. Moretti

**Affiliations:** ^1^Department of Dermatological Sciences, University of Florence, 50121 Florence, Italy; ^2^Department of Clinical, Preventive, and Oncologic Dermatology, University of Florence, Villa Basilewsky, Via Lorenzo Il Magnifico 104, 50129 Florence, Italy; ^3^Department of Critical Care Medicine and Surgery, University of Florence, Villa Basilewsky, Via Lorenzo Il Magnifico 104, 50129 Florence, Italy

## Abstract

The incidence of melanoma, the most aggressive type of cutaneous malignant tumor, is currently on the rise. Treatment in advanced stages is still unsuccessful compared with other malignant tumors, thus it is important to indentify the key mechanisms responsible for melanoma progression and metastasis. Genetic and molecular components, in particular, that are up- or downregulated in melanoma cells, affect the invasive potential of melanoma. Another possible important cofactor highlighted by recent studies is chronic stress, involving environmental and psychological factors, which can be an important cofactor in not only cancer progression in general but also in melanoma spreading. The negative effects of chronic stress have been evaluated epidemiologically in patients with breast and prostate cancer. In particular, the effects of stress mediators, namely, catecholamines have been studied on various human malignancies, including melanoma and have highlighted a significant increase of progression-related molecules. As such, this could be the starting point for a new approach in the treatment of advanced melanoma, in which the negative effects of stress are reduced or blocked.

## 1. Introduction

At present, melanoma remains the most aggressive type of cutaneous malignant tumor. In contrast to the incidence of other tumors, the incidence of melanoma continues to increase, the majority of which is seen in young adults. Consequently, death attributed to melanoma is higher than most other solid tumors [1].

Malignant melanoma has had a rapid increase in incidence in recent years, without being associated with better therapeutic options. Compared to other malignant tumors that can be treated effectively even in advanced stages, metastatic melanoma still has a poor response to chemotherapy, immunotherapy, and radiation therapy. At present, surgery in the early stages represents the only approach to quickly eradicate the disease [2]. It is crucial moreover to prevent or reduce the factors capable of affecting melanoma progression and diffusion, due to the intractability of advanced melanoma. To do so, we must understand the key aspects concerning the mechanisms underlying local and distant invasion of this malignant tumor.

## 2. Main Factors Affecting Melanoma Progression

The progression of melanoma is affected by a series of genetic and molecular mechanisms. Transition from normal melanocytes to malignant metastatic cells is the consequence of up- and downregulation of complex cellular processes [3]. One of the most important and studied invasion mechanisms of melanoma cells is the expression of various adhesion membrane molecules.

Physiologically, epidermal melanocytes are connected with keratinocytes through multiple contacts between the cells. In this manner, keratinocytes control melanocyte growth and cell surface receptor expression. Melanoma cells escape this control mechanism and begin to invade the dermis through a number of mechanisms implemented by melanoma cells themselves. 

Adhesion molecules, such as E-cadherin, P-cadherin, and desmoglein, result in being downregulated in melanoma cells which can disengage from keratinocytes [3]. Instead, N-cadherin, Mel-CAM, and zonula occludens protein-1 are upregulated on melanoma cells, facilitating interaction with stromal fibroblasts [4] and endothelial cells and allowing entry into the vasculature [5]. That is to say, during melanoma progression, the loss of E-cadherin expression [6] disrupts normal homeostasis by freeing melanoma cells from structural and functional regulation by keratinocytes and is paralleled by a gain in N-cadherin function [4] that mediates homotypic interaction between melanoma cells, facilitates gap-junctional formation with fibroblasts and endothelial cells, and promotes melanoma cell migration and survival [7].

The integrin family also plays a role in regulating other processes involved in progression and metastasis of melanoma. Integrins are adhesion molecules that couple the extracellular environment to the cytoskeleton, while also transmitting intracellular signals responsible for an assortment of cellular processes including survival, migration, invasion, and proliferation [7, 8]. In particular, the integrin family member *α*v*β*3 [9] seems to be widely expressed on melanoma cells in the vertical growth phase and is associated with increased tumor growth in vivo. Thus during melanomagenesis, melanocytes show increased levels of *α*v*β*3 integrin concomitant with the loss of E-cadherin expression [10]. 

Moreover, tissue invasion is mediated by proteinases specific for interstitial extracellular matrix such as metalloproteinases (MMPs), particularly matrix metalloproteinases MMP-2 and MMP-9. The MMPs belong to a family of calcium- and zinc-dependent endopeptidase and can digest a wide range of matrix extracellular molecules. In fact the MMPs are implicated in tumor cell invasion through the degradation of interstitial and basement membrane extracellular matrix. It is this event that represents an important stage of tumor progression and is demonstrated principally by a coexpression of MMP-2 and MMP-9 in invasive and in situ melanoma [11]. 

Diffusion and progression are further influenced by autocrine and paracrine production of cytokines [12] such as, for example, Transforming Growth Factor (TGF) beta, Hepatocyte Grow Factor (HGF), Fibroblast Growth Factor (FGF), Vascular Endothelial Growth Factor (VEGF) A, and Vascular Endothelial Growth Factor (VEGF) C. These growth factors are very important for melanoma progression, because they support cell survival and enhance metastatic potential, creating a microenvironment that promotes growth and tumor expansion [13, 14]. In particular, VEGF is an essential factor for tumor angiogenesis that increases the permeability of microvessels, acts as a selective endothelial cell mitogen, and induces increased production of other tissue factors and several proteases [11]. These events are confirmed by data that show that malignant melanocytic tumors displayed strong VEGF expression whereas benign melanocytic proliferations showed no immunoreactivity for VEGF [11].

Changes in migration, invasion, proliferation, and survival [7, 8, 11, 13, 14] are responsible for the invasive potential of melanoma, and in vitro studies show that multiple mechanisms are involved in these processes besides the above mentioned, and they include extension of protrusions (induced by actin polymerization) [15, 16], contraction (a process actin-myosin linked) deadhesion (a mechanism mediated by actin disassembly [17]), sliding, and fragmentation of the cell [18–21].

## 3. Stress and Cancer

Significant evidence exists demonstrating that chronic stress and cancer progression are connected. To begin with, stress leads to an important activation in both central and peripheral nervous systems and increased hormone and peptide production (particularly neuropeptides and neurotransmitters). These events are considered physiological and adaptive responses in acute stress but are harmful in chronic stress conditions [22]. Therefore chronic stress, defined as a complex process including environmental and psychological factors, can influence not only cellular immune parameters with a reduction in immune response but can also influence other biological pathways. Recent studies have thoroughly investigated these effects, evaluating the effects of stress on the course of some malignant tumors [22]. Epidemiological data show that chronic depression due to separation, divorce, or death of spouse may have an important role in the onset, progression, and response to breast cancer therapy. In fact, a US breast cancer study demonstrated that the risk of developing breast cancer increases if stress conditions and depression persist for at least six years [23] and that the mood of the patients seems to be very important in disease progression. Patients with a negative reaction to cancer diagnosis have a faster disease progression and a poorer response to therapy. Conversely, “positive" factors, such as the presence of social support and an optimistic attitude toward illness, favor a longer survival [23]. Experimental stressors have also been found to increase the pathogenesis of various virus-mediated tumors in animal models, such as leukemia, lymphoma, liver carcinoma, Kaposi-sarcoma, cervical cancer, and rectal cancer [24] In hepatocellular carcinoma, animals were treated with the carcinogen diethylnitrosamine associated with immobilization that increased both incidence and rate of tumor growth [25], confirming the aforementioned results. 

An additional study on mice by Hasegawa and Saiki [26] demonstrated the presence of an interrelationship among psychosocial stress, tumor development, and beta-adrenergic activation. The application of several stress paradigms including rotation, social housing conditions, and restraint enhanced tumor growth of B16 melanoma implanted in the footpad of syngeneic male mice. It has been also demonstrated that chronic behavioural stress results in high levels of tissue catecholamines, greater tumor burden, and more invasive growth of ovarian carcinoma cells in an orthotopic mouse model. Tumors in stressed animals showed markedly increased vascularization and enhanced expression of VEGF, MMP-2, and MMP-9; it seems that angiogenic processes mediate the effects of stress on tumor growth in vivo [27]. 

With regard to human cancer, a study by Sood et al. [28] on human ovarian carcinoma cells has demonstrated that chronic behavioural stress and, above all, the neuroendocrine corresponding responses can affect ovarian cancer cell biology, enhancing angiogenesis and tumor growth. This effect is mediated by catecholamine interaction with beta2-adrenergic receptors expressed on ovarian tumor cells that can influence tumor progression by stimulating the expression of MMPs. It has further been demonstrated that exposure to epinephrine and norepinephrine increases ovarian cancer cell invasiveness by 89% to 198%, and these effects are completely abolished by adrenergic beta-blockers such as propranolol [28–30]. The MMPs (particularly MMP-2 and MMP-9) and VEGF expression has been studied also in three nasopharyngeal carcinoma human cell lines as factors that can contribute to tumor development and aggressiveness under stimulation of catecholamines. Yang et al. have shown that in nasopharyngeal carcinoma cells expressing beta2-adrenergic receptor, these hormones induce the production of all three molecules, which are blocked by beta-adrenergic antagonists such as propranolol [31].

Recently, Yang and colleagues have studied the possible role of catecholaminergic stimulation on human multiple myeloma cells, in order to demonstrate if VEGF is differentially regulated by norepinephrine. The authors have confirmed the presence of beta1- and beta2-adrenergic receptors on three different myeloma cell lines and demonstrated that norepinephrine was able to enhance the production of VEGF. These data indicate that angiogenesis is stimulated by catecholamines also in multiple myeloma and that stress is an important mechanism in the biology and progression of various cancers [32].

## 4. Melanoma and Stress

Melanoma cells also express both alpha- and beta adrenoreceptors, which can be activated by catecholamines released under stress. Shih and Lo in 1993 [33] conducted a study which pointed that GMM-1 cells (a goldfish melanocytoma cell line) treated with epinephrine exhibited a rapid expansion. This effect consisted in various changes of cellular morphology, namely, flattening of cells and extension and broadening of cellular processes, which suggest that the effects of various epinephrine agonists and antagonists may be caused by multiple isoforms of adrenoceptors in goldfish cells [33].

Scarparo et al. have demonstrated the presence of low affinity alpha1-adrenoceptors in SK-Mel 23 human melanoma cells. Alpha1-adrenoceptors are coupled to Gq protein, and they activate phospholipase C (PLC) and increase intracellular calcium. When activated by catecholamines they mediate a decrease in cell proliferation and an increase in tyrosinase activity, with no change of tyrosinase expression. The administration of an alpha1-adrenergic agonist such as phenylephrine thereby induces decreased proliferation and increased tyrosinase activity while an alpha1-adrenergic antagonist such as prazosine blocks these activities. It therefore seems that alpha1-adrenoceptors, expressed by melanoma cells, have a low affinity for catecholamines and exert a marginal role in tumor growth and invasion [34, 35]. 

Recently, Yang et al. [36] investigated the expression of beta1- and beta2-adrenergic receptors in three cell lines of human melanoma, C8161, 1174MEL, and Me18105 and the expression of both adrenoceptors, and analyzed the cellular response to norepinephrine stimulation. The presence of beta1- and beta2-adrenoceptors was assessed in primary and metastatic human melanoma cells by immunohistochemistry. Norepinephrine was able to enhance tumor progression, by stimulating the secretion of factors implicated in angiogenesis and metastasis [36]. 

In particular, melanoma cells treated with norepinephrine exhibited an upregulation of VEGF, IL-8, and IL-6. These three cytokines play proangiogenic, chemotactic, or autocrine stimulant activity, respectively, and are closely related with melanoma progression, considering that their production by melanomatous cells is increased in advanced tumor stages [14, 36].

These data are of particular interest because they support the hypothesis that catecholamines, which are typical stress hormones, can promote the aggressive potential of melanoma tumor cells through the interaction with specific receptors. Intervention targeting these receptors or the production of stress hormones, such as pharmacological treatment with adrenoceptor-blocking agents or social and psychological support, may represent a valid approach in the treatment of advanced melanoma, which is at present an unresponsive disease [36] and potentially even in early stages of the disease.

## References

[1] MacKie RM, Hauschild A, Eggermont AM (2009). Epidemiology of invasive cutaneous melanoma. *Annals of Oncology*.

[2] Atallah E, Flaherty L (2005). Treatment of metastatic malignant melanoma. *Current Treatment Options in Oncology*.

[3] Haass NK, Herlyn M (2005). Normal human melanocyte homeostasis as a paradigm for understanding melanoma. *The Journal of Investigative Dermatology. Symposium Proceedings *.

[4] Smalley KSM, Brafford P, Haass NK, Brandner JM, Brown E, Herlyn M (2005). Up-regulated expression of zonula occludens protein-1 in human melanoma associates with N-cadherin and contributes to invasion and adhesion. *American Journal of Pathology*.

[5] Bogenrieder T, Herlyn M (2002). Cell-surface proteolysis, growth factor activation and intercellular communication in the progression of melanoma. *Critical Reviews in Oncology/Hematology*.

[6] Hsu M-Y, Wheelock MJ, Johnson KR, Herlyn M (1996). Shifts in cadherin profiles between human normal melanocytes and melanomas. *Journal of Investigative Dermatology Symposium Proceedings*.

[7] Johnson JP (1999). Cell adhesion molecules in the development and progression of malignant melanoma. *Cancer and Metastasis Reviews*.

[8] Bauer EA, Uitto J, Walters RC, Eisen AZ (1979). Enhanced collagenase production by fibroblasts derived from human basal cell carcinomas. *Cancer Research*.

[9] Seftor REB (1998). Role of the *β*
_3_ integrin subunit in human primary melanoma progression: multifunctional activities associated with *α*
_v_
*β*
_3_ integrin expression. *American Journal of Pathology*.

[10] Albelda SM, Mette SA, Elder DE (1990). Integrin distribution in malignant melanoma: association of the *β*
_3_ subunit with tumor progression. *Cancer Research*.

[11] Simonetti O, Lucarini G, Brancorsini D (2002). Immunohistochemical expression of vascular endothelial growth factor, matrix metalloproteinase 2, and matrix metalloproteinase 9 in cutaneous melanocytic lesions. *Cancer*.

[12] Lázár-Molnár E, Hegyesi H, Tóth S, Falus A (2000). Autocrine and paracrine regulation by cytokines and growth factors in melanoma. *Cytokine*.

[13] Al-Alousi S, Carlson JA, Blessing K, Cook M, Karaoli T, Barnhill RL (1996). Expression of basic fibroblast growth factor in desmoplastic melanoma. *Journal of Cutaneous Pathology*.

[14] Moretti S, Pinzi C, Spallanzani A (1999). Immunohistochemical evidence of cytokine networks during progression of human melanocytic lesions. *International Journal of Cancer*.

[15] Kovacs EM, Makar RS, Gertler FB (2006). Tuba stimulates intracellular N-WASP-dependent actin assembly. *Journal of Cell Science*.

[16] Nakahara H, Otani T, Sasaki T, Miura Y, Takai Y, Kogo M (2003). Involvement of Cdc42 and Rac small G proteins in invadopodia formation of RPMI7951 cells. *Genes to Cells*.

[17] Ezratty EJ, Partridge MA, Gundersen GG (2005). Microtubule-induced focal adhesion disassembly is mediated by dynamin and focal adhesion kinase. *Nature Cell Biology*.

[18] DesMarais V, Ghosh M, Eddy R, Condeelis J (2005). Cofilin takes the lead. *Journal of Cell Science*.

[19] Seftor REB, Seftor EA, Hendrix MJC (1999). Molecular role(s) for integrins in human melanoma invasion. *Cancer and Metastasis Reviews*.

[20] Dang D, Bamburg JR, Ramos DM (2006). *α*
_v_
*β*
_3_ integrin and cofilin modulate K1735 melanoma cell invasion. *Experimental Cell Research*.

[21] Fukata Y, Amano M, Kaibuchi K (2001). Rho-Rho-kinase pathway in smooth muscle contraction and cytoskeletal reorganization of non-muscle cells. *Trends in Pharmacological Sciences*.

[22] Thaker PH, Lutgendorf SK, Sood AK (2007). The neuroendocrine impact of chronic stress on cancer. *Cell Cycle*.

[23] Penninx BWJH, Guralnik JM, Pahor M (1998). Chronically depressed mood and cancer risk in older persons. *Journal of the National Cancer Institute*.

[24] Antoni MH, Lutgendorf SK, Cole SW (2006). The influence of bio-behavioural factors on tumour biology: pathways and mechanisms. *Nature Reviews Cancer*.

[25] Laconi E, Tomasi C, Curreli F (2000). Early exposure to restraint stress enhances chemical carcinogenesis in rat liver. *Cancer Letters*.

[26] Hasegawa H, Saiki I (2002). Psychosocial stress augments tumor development through *β*-adrenergic activation in mice. *Japanese Journal of Cancer Research*.

[27] Thaker PH, Han LY, Kamat AA (2006). Chronic stress promotes tumor growth and angiogenesis in a mouse model of ovarian carcinoma. *Nature Medicine*.

[28] Sood AK, Bhatty R, Kamat AA (2006). Stress hormone-mediated invasion of ovarian cancer cells. *Clinical Cancer Research*.

[29] Skilling JS, Squatrito RC, Connor JP, Niemann T, Buller RE (1996). p53 Gene mutation analysis and antisense-mediated growth inhibition of human ovarian carcinoma cell lines. *Gynecologic Oncology*.

[30] Sood AK, Seftor EA, Fletcher MS (2001). Molecular determinants of ovarian cancer plasticity. *American Journal of Pathology*.

[31] Yang EV, Sood AK, Chen M (2006). Norepinephrine up-regulates the expression of vascular endothelial growth factor, matrix metalloproteinase (MMP)-2, and MMP-9 in nasopharyngeal carcinoma tumor cells. *Cancer Research*.

[32] Yang EV, Donovan EL, Benson DM, Glaser R (2008). VEGF is differentially regulated in multiple myeloma-derived cell lines by norepinephrine. *Brain, Behavior, and Immunity*.

[33] Shih Y-L, Lo SJ (1993). Induction of cell expansion of goldfish melanocytoma cells (GMM-1) by epinephrine and dexamethasone requires external calcium. *Cell Biology International*.

[34] Scarparo AC, Sumida DH, Patrão MTCC, Avellar MCW, Visconti MA, Castrucci AMDL (2004). Catecholamine effects on human melanoma cells evoked by *α*1-adrenoceptors. *Archives of Dermatological Research*.

[35] Scarparo AC, Visconti MA, de Lauro Castrucci AM (2006). Signalling pathways evoked by *α*
_1_-adrenoceptors in human melanoma cells. *Cell Biochemistry and Function*.

[36] Yang EV, Kim S-J, Donovan EL (2009). Norepinephrine upregulates VEGF, IL-8, and IL-6 expression in human melanoma tumor cell lines: implications for stress-related enhancement of tumor progression. *Brain, Behavior, and Immunity*.

